# Assessment of health risks associated with heavy metal concentration in seafood from North-Western Croatia

**DOI:** 10.1038/s41598-023-43365-7

**Published:** 2023-09-29

**Authors:** Luka Traven, Sandra Marinac-Pupavac, Paula Žurga, Željko Linšak, Sandra Pavičić Žeželj, Marin Glad, Darija Vukić Lušić

**Affiliations:** 1 Department of Environmental Medicine, Medical Faculty, University of Rijeka, Braće Branchetta 20/1, Rijeka, Croatia; 2 Teaching Institute of Public Health, Krešimirova 52a, 51000 Rijeka, Croatia

**Keywords:** Biotechnology, Health care, Risk factors

## Abstract

The following study aims at assessing the health risks associated with the consumption of the most commonly consumed seafood in the north-western part of Croatia due to the presence of heavy metals. Samples of seafood were collected and analysed for lead (Pb), cadmium (Cd), and mercury (Hg) content. Analyses of Cd and Pb were carried out by inductively coupled plasma mass spectrometry (ICP-MS) whereas Hg content was measured using atomic absorption spectrometry (AAS). Metal concentrations were in the following order Hg > Pb > Cd for the gilthead seabream, European hake, sardines, and tuna fish whereas in the Patagonian squid cadmium (Cd) was the heavy metal with the highest concentration, with the order of other metals being Cd > Hg > Pb. The heavy metal concentrations have been used to address the health risks using the Estimated Weekly Intake (EWI), Target Hazard Quotients (THQ), and Hazard Indices (HI). The findings revealed that the concentrations of the tested heavy metals, expressed on a per wet weight basis, did not exceed the Maximum Residue Levels (MRL) for those compounds mandated by national Croatian legislation. However, the HI for Hg was above 1, indicating a risk of adverse health effects due to the presence of this heavy metal in the consumed seafood. We conclude that the consumption of certain type of seafood such as the tuna fish should be limited when sensitive segments of the population such as children, elderly and pregnant women are concerned. Our results strongly advocate for a more stringent seafood quality control in the region.

## Introduction

A balanced and healthy human diet should include at least 1–2 servings of fish and other seafood per week^1–3^. Seafood is rich in both macro and micronutrients, which are important in the prevention of malnutrition and diet-related chronic diseases^4^. In addition, fish is rich in fatty acids having very strong cardioprotective properties, including docosahexaenoic (DHA) and eicosapentaenoic (EPA) fatty acids^5,6^.

Industrial development has led to heavy metals being emitted into the aquatic environment where they can persist for very long periods of time^7,8^. In general, heavy metals are deposited in marine sediments and enter the food web via benthic organisms. Due to their potential to bioaccumulate, the concentration of heavy metals in organisms located at higher trophic levels can reach concentrations that may be harmful to human health. Thus, the consumption of seafood in general and fish in particular is the main exposure route to heavy metals^9–11^. Exposure to heavy metals including lead (Pb), cadmium (Cd) and mercury (Hg) can lead to a number of adverse health effects, including a compromised renal and hepatic function^12^, teratogenic effects^13–15^, and cognitive impairments^16,17^.

Besides fish, the presence of heavy metals has been detected in other taxonomic groups as well. In the study by Nigariga et al.^18^ the presence of heavy metals has been identified in edible brown, red and green algae in southeast India. Although Cd, Zn, Pb and Cu were present in the species tested, the levels did not exceed the maximum allowable levels for human consumption. Regarding mussels (*Mytillus galloprovincialis*), the presence of Cd, Pb, Hg and As were detected in mussels across different regions of the Mediterranean Sea with wide variations in concentrations^19^. The heavy meal concentration in the tested samples were in the following order: As > Pb > Cd > Hg. The study by Yabanli et al.^20^ has also found the presence of heavy metals in the tissue of mussels, with the reported values (mg kg^−1^ of dry weight) as follows: 0.17 and 0.15 for Cr, 28.62 and 29.49 for Fe, 0.25 and 0.29 for Ni, 2.53 and 1.78 for Cu, 18.52 and 22.03 for Zn, 1.26 and 1.08 for As, 0.04 and 0.04 for Cd, 0.02 and 0.02 for Hg, 0.19 and 0.16 for Pb, 0.40 and 0.48 for Se. In addition, it has been proven that the presence of heavy metals can cause proteosome-mediated protein degradation in crab (*Cancer paragus*) but the effect was smaller compared to other species of crustaceans such as lobsters^21^. Regarding fish, the study by Gu et al.^22^ analysed heavy metal concentrations in 29 marine wild fish species from the South China Sea. Concentrations expressed on a per wet weight basis were as follows: 0.51–115.81 ng/g (Cd), 0.54–27.31 ng/g (Pb), 0.02–1.26 μg/g (Cr), 8.32–57.48 ng/g (Ni), 0.12–1.13 μ/g (Cu), 2.34–6.88 μg/g (Zn), 2.51–22.99 μ/g (Fe), and 0.04–0.81 μ/g (Mn). Interestingly, for some fish species Fe and Mn concentrations were above the acceptable daily upper limit, suggesting an increased health risk due to the consumption of these species of fish.

In Croatia, the maximum level of heavy metals in food, including seafood, is regulated under the Regulation on the Maximum Permissible Levels of Certain Contaminants in Food^23^ which, among others, specifies the Maximum Residue Levels of heavy metals in seafood. Even though the concentration of heavy metals in seafood is regulated, academic studies often report levels of heavy metals in seafood which exceed the legal limits^24^.

Because individual metals vary in toxicity, the Maximum Residue levels (MRLs) and Provisional Tolerable Weekly Intake (PTWI) for metals in seafood are specified for each heavy metal. The MRLs indicate limits above which consumers will be exposed to harmful levels of the contaminant, while the PTWI indicates the tolerable weekly human exposure to metal contaminants associated with food consumption^25^.

Although levels of heavy metals in seafood have been reported previously in Croatia^26^, to our knowledge the characterization of health risks posed by traditional heavy metals (Cd, Pb and Hg) due to the consumption of seafood have not been yet performed for this region. We believe this study to be of paramount importance as it sheds light on the critical issue of seafood consumption in the north-western part of Croatia, particularly emphasizing the health risks due to the presence of heavy metals in seafood such as Pb, Cd, and Hg. The study revealed that Hg had a hazard index (HI) above 1, indicating an increased risk to human health due to the consumption of seafood. We believe that the results of this study will be informative for both the academic community and domain experts as well as regulatory agencies such as the Croatian agency for Agriculture and Food and the European Food Safety Authority (EFSA).

## Materials and methods

### Sampling location and collection of samples

**Figure 1:**
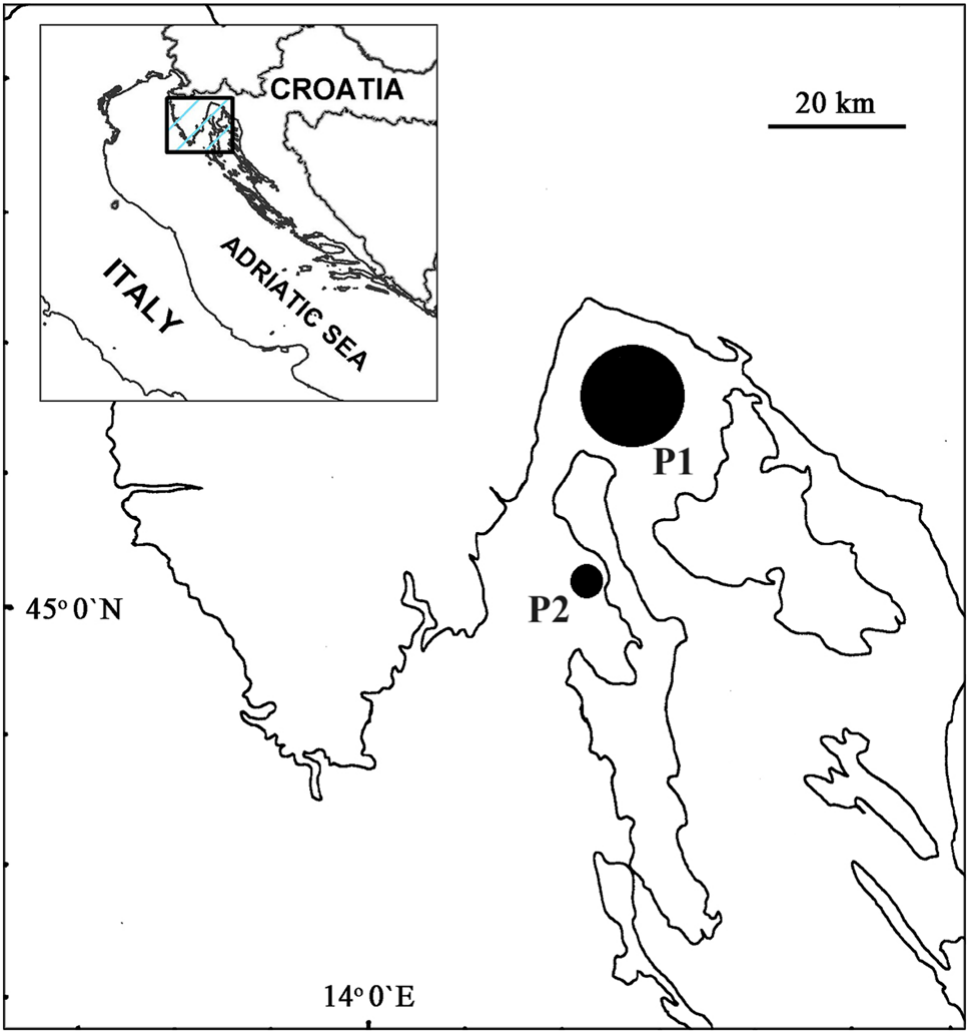
Sampling locations. Europanean hake, sardines and tuna fish were fished at location P1, whereas the gilthead seabream has been farmed at location P2. Tuna fish has been farmed in Italy whereas the Patagonian squid has been obtained from the Atlantic Ocean.

The Primorje and Gorski Kotar county is located in the north-western part of the Republic of Croatia. It includes the Kvarner Bay, the Norther Croatian littoral as well as the inland region of Gorski Kotar (Fig. 1 upper corner). The sampling of seafood has been performed at local markets in the region, whereas the fishing location for fish obtained from the Adriatic sea (Croatian part) is shown in Fig. 1. Tiuna fish has been farmed in Italy whereas the Patagonian squid were obtained from the Atlantic Ocean (data not shown). The seafood was obtained from local markets.

Seafood consumed in the region is mostly bought fresh from local markets. Samples of gilthead seabream (*Sparus aurata*, L), European hake (*Merluccius merluccius*, Linnaeus 1758), sardines (*Sardinia pilchardus*, Walbaum, 1792), tuna fish (*Thunnus thynnus*, Linnaeus 1758) and Patagonian squid (*Loligo gahi*, Orbigny 1835) have been obtained from the local markets during a sampling campaign which lasted from February 2022 until April 2022. In total, 60 samples have been collected, twelve samples per species. The samples consisted of a different number of individuals from which composite samples were prepared. The number of individuals in each composite sample is shown in Table 1.

**Table 1:** Number of individuals per composite sample.

Species	n of individuals per composite sample
Gilthead seabream	2
European Hake	3–7
Sardine	9–12
Tuna fish	2
Patagonian squid	9–12

Sardines and the European hake were fished in Croatia, in the Mediterranean Sea, whereas the gilthead seabreams were obtained from local fish farms. Tuna fish was farmed in Italy and the Patagonian squid has been caught in the Atlantic Ocean. After collection, the samples were transported on ice to the laboratory. The samples have been stored at − 20 °C until further processing. For each sample a single composite sample has been prepared. Before the analyses the samples were lyophilized (apparatus: LIO-5 PLT) and pulverized in a cryogenic mill (MM 500 control, Retsch).

### Analyses of heavy metals

Freeze-dried subsamples of composites were microwave digested (Anton Paar Multivawe 3000, Perkin Elmer, USA) in 65% nitric acid (HNO3, Suprapur, Merck, Germany). Digested samples were diluted with ultrapure water (Ultra Clear, Siemens) and analysed for Pb, Cd and Hg. Analyses Cd and Pb were carried out with inductively coupled plasma mass spectrometer (ICP MS NexION 300X) equipped with S10 autosampler (Perkin Elmer, USA). Analyses of Hg were performed by atomic absorption spectrometry (AMA 254, Advanced Mercury Analyser, Leco, USA).

### Risk assessment

The health risk assessment has been performed using the following indicators: the Estimated Weekly Intake (EWI), the Provisional Tolerable Weekly Intake (PTWI), the Target Hazard Quotient (THQ) and the Hazard Index (HI).

#### Consumption of seafood

Weekly consumption of seafood—412.3 g.

Patagonian squid—30.8 g/day.

Tuna fish—12.9 g/day.

Sardines—12.9 g/day.

European hake—16.6 g/day.

Giltehead seabream—16.6 g/day.

The data on seafood consumption were obtained from Marinac et al.^27^.

#### Estimated weekly intake (EWI)

The estimated weekly intake was calculated according to the following formula:

1 $$EWI = { }\frac{C*Cons}{{BW}}$$

where C—concentration of the heavy metals (mg/kg). Cons—average weekly consumption of seafood (kg). BW—average body weight (70 kg)^28^.

#### Target hazard quotient (THQ)

The THQ has been calculated according to the following formula:

2 $$TGQ = \frac{EF*ED*FIR*C}{{RfD*BW*TA}}*10^{ - 3}$$

where THQ—target hazard quotient. EF—exposure frequency (365 days/year). ED—exposure duration (70 years). FIR—seafood ingestion rate (g/day). C—metal concentration in seafood tissue (mg/kg). RfD—oral reference dose (mg/kg day). BW—average body weight (70 kg). TA—exposure time for non-carcinogens (365 days/year ED).

#### Hazard index (HI)

The HI has been calculated according to the following formula:

3 $$HI = \sum THQ$$

where HI—hazard index. THQ—target hazard quotients for each heavy metal.

### Quality assurance

For analytical quality assurance, appropriate blanks and certified reference material has been used. The analyses were performed in triplicates. The method for the measurement of Cd and Pb has been validated with IAEA-407 (fish tissue; International Atomic energy Agency, Austria) with the mean recovery between 89 and 110%. For the validation of Hg measurements (mean recovery 104%) the NIST 2976 (mussel tissue, National Institute of Standards and Technology, USA) has been used.

### Statistical analyses

The distribution of data sets has been tested with the Anderson-Darling test (α = 0.05). If the data did not follow a normal distribution, nonparametric statistical tests were used. The address whether the sample median differed significantly from the Maximum Residual Value (MRL) the Wilcoxon signed rank test has been used. To test the differences between groups the Kruskal-Wallis test has been used followed by the Dunn’s post-hoc test. All the analyses have been performed using GraphPad Prism version 9.0.0 for Windows, GraphPad Software, San Diego, California USA, www.graphpad.com.

## Results and discussion

### Concentration of heavy metals in seafood

In this study the concentrations of Pb, Cd and Hg in 60 composite samples of seafood most frequently consumed in the north-western part of Croatia have been studied and the health risk associated with seafood consumption due to the presence of these heavy metals have been calculated. The species tested included the following: gilthead seabream, European hake, sardines, Patagonian squid, and tuna fish.

Pb has been detected in 28%, Cd in 59% and Hg in all analysed samples (100%). Metal concentrations were in order Hg > Pb > Cd for the gilthead seabream, European hake, sardines, and tuna fish whereas in the Patagonian squid Cd was the heavy metal with the highest concentration with the order being Cd > Hg > Pb. None of the tested samples where above the Maximum Residue Level (MRL) mandated by the Croatian Regulation on the Maximum Permissible Levels of Certain Contaminants in Food^23^. Descriptive statistics of lead (Pb), cadmium (Cd) and Hg (Cd) in the samples tested are shown in Table 2.

**Table 2:** Descriptive statistics for the heavy metals measured in this study.

Species	Heavy metal (μg/kg d.w.)	n of composite samples	MRL (µg/kg d.w.)	Median (μg/kg d.w.)	95% CI of the median (µg/kg d.w.)	Range (μg/kg d.w.)
Gilthead seabream	Pb	12	0.30	0.008	0.008–0.008	0.007–0.060
	Cd	12	0.05	< 0.01		
	Hg	12	0.50	0.038	0.037–0.0.41	0.030–0.10
European hake	Pb	12	0.30	< 0.025		
	Cd	12	0.05	< 0.01		
	Hg	12	0.50	0.142	0.130–0.176	0.115–0.276
Sardine	Pb	12	0.30	0.014	0.012–0.016	0.005–0.127
	Cd	12	0.25	0.005	0.004–0.005	0.002–0.017
	Hg	12	0.50	0.066	0.062–0.68	0.055–0.094
Tuna fish	Pb	12	0.30	< 0.025		
	Cd	12	0.10	0.006	0.004–0.009	0.002–0.032
	Hg	12	1.00	0.370	0.225–0.469	0.138–1.135
Patagonian squid	Pb	12	0.30	< 0.025		
	Cd	12	1.00	0.545	0.464–0.640	0.34–0.899
	Hg	12	0.50	0.028	0.025–0.29	0.009–0.032

Although we did not find concentrations of metals exceeding the legal limits, heavy metal concentrations more than the legal limits are often reported by academic studies in the region^24,29,30^. The fact that heavy metals often exceed the legal limits is not surprising since the adherence to legal limits and overall food safety is the responsibility of the food producer and consequently the number of samples tested is usually small, which often leads to false negative results. Although the legal limit for tested heavy metals was not exceeded, the HI for Hg in seafood consumed was above 1 which strongly argues in favour of a more stringent seafood quality control in Croatia in general, and the north-western part in particular.

**Figure 2:**
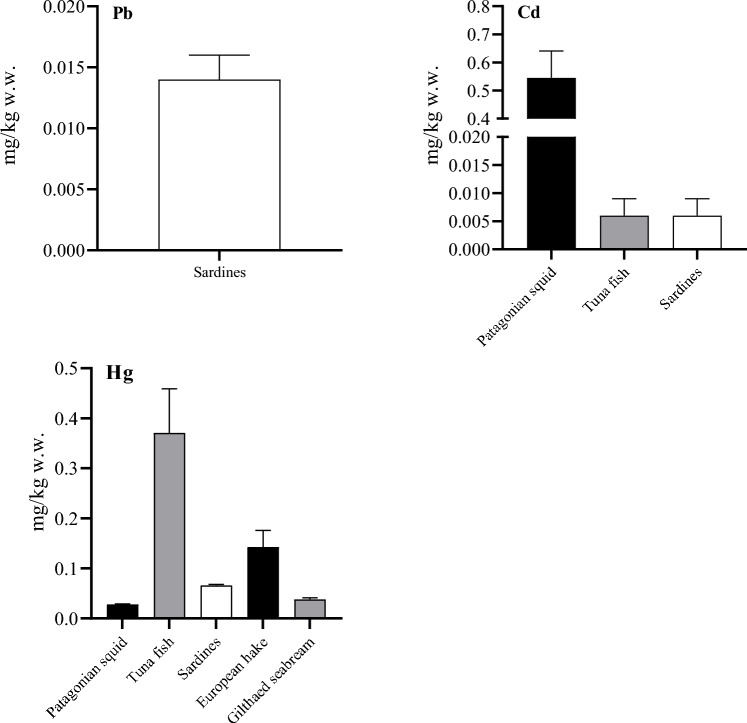
Comparison of heavy metal concentrations (Pb, Cd, Hg) between he tested species.

Regarding the difference in heavy metal content between the tested species, the cadmium content in Patagonian squid was significantly higher than in other species tested (p < 0.0001), whereas the mercury content was significantly higher in tuna fish and the European hake compared to other tested species (p < 0.0001). The median value of the heavy metals tested with a 95% CI interval of the median for each tested species is shown in Fig. 2.

The fact that European hake and tuna fish had a high Hg content should not be surprising considering that Hg exhibits a strong bioaccumulation and biomagnification potential^31–33^. Regarding the high Cd content in the Patagonian squid, we speculate that this is the result of the fact that the Patagonian squid has been fished from environments with comparably high Cd concentrations. Cadmium may passively diffuse into marine organisms or enter the marine food chain at the level of plankton and microorganisms and then enter fish via food. In addition, according to Kojadinović et al.^34^ and Bustamante et al.^35^ cephalopods often express high levels of Cd content due to the essential role that this heavy metal plays in physiological and detoxification processes. Pb levels were comparably low, probably due to a reduction in anthropogenic lead pollution which has a direct link to the marine environment, which acts as a sink for Pb. Thus, a reduction in anthropogenic lead emissions results in low levels of lead in both seawater and marine organisms^36^. Overall, the concentrations of heavy metals found in this study are in good agreement with values reported by studies performed in other European countries^37–40^.

Table 3 compares the on heavy metal concentration in seafood from different regions.

**Table 3:** Concentrations of lead (Pb), cadmium (Cd), and mercury (Hg) (mean ± s.d., µg/g w.w.) in seafood in different locations in the world.

Location	Pb (mg/kg w.w.)	Cd (mg/kg w.w.)	Hg (mg/kg w.w.)	References
North-East Mediterranean Sea	0.91 ± 0.08	< 0.0004		Korkmaz et al.^41^
	0.57–1.37			
	0.81 ± 0.32	< 0.0004		Korkmaz et al.^42^
	(< 0.003–1.25)			Licata et al.^43^
Central Mediterranean Sea	0.02 ± 0.06	0.03 ± 0.07	3.03 ± 0.55	
	(n.d.–0.24)	(n.d.–0.26)	(2.45–4.1)	
	0.31 ± 0.35	1.19 ± 0.46	1.88 ± 0.54	
	(0.06–1.29)	(0.38–1.75)	(1.32–3,02)	
The northern Levantine Sea	0.115 ± 0.159	0.002 ± 0.003	0.001 ± 0.000	Mol et al.^44^
	(0.000–0.912)	(0.000–0.0012)	(0.000–0.002)	
Adriatic Sea	0.021 ± 0.008	0.008 ± 0.002	0.142 ± 0.024	Jureša et al.^45^
	0.023 ± 0.007	0.007 ± 0.002	0.20 8 ± 0.037	
	0.007 ± 0.004	0.002 ± 0.001	0.275 ± 0.117	
	0.023 ± 0.002	0.002 ± 0.001	0.373 ± 0.075	
Western Atlantic Ocean		0.005 ± 0.002	0.021 ± 0.004	Nyarko et al.^46^

Range of concentrations is indicated in parenthesis (where available). nd—non detectable.

### Characterization of health risks

#### Estimated weekly intake (EWI)

The estimated weekly intake (EWI) and the percentage of the Provisional Tolerable Weekly Intake (PTWI) for Pb, Cd and Hg as given by FAO/WHO^47^ are shown in Table 4.

**Table 4:** The estimated weekly intake and the provisional tolerable weekly intake for the heavy metals studied.

	Pb (μg/kg)	Cd (μg/kg)	Hg (μg/kg)
Estimated weekly intake (EWI)	1.76	0.07	1.07
Provisional tolerable weekly intake (PTWI)	25	7	1.6
% PTWI	7.04%	0.95%	67.06%

As it can be seen from Table 3, the estimated weekly intake from Cd and Hg did not exceed the threshold value given by the Tolerable Weekly intake. Thus, according to the EWI, the population of the north-western part of Croatia does not have heightened risk of adverse effects due to the exposure to heavy metals via seafood consumption.

#### Target hazard quotients (THQ)

In addition to the EWI, health risks related to heavy metal concentrations in seafood have been addressed using the target hazard quotients (THQ) as well as the hazard index (HI). The Target Hazard Quotient (THQ) represents the quotient between the exposure to a pollutant and the acceptable level of that pollutant below which no adverse effects are to be expected.

#### Hazard index

The hazard index (HI), on the other hand, is the sum of the THQ values for each heavy metal. In other words, the HI provides an assessment of the health risks resulting from an exposure to a mixture of heavy metals. It is generally accepted that a HI value greater than 1.0 indicates the possibility of adverse health effects. The target hazard quotients (THQ) and the Hazard Index were estimated based on the consumption seafood in the region as described in our previous work ^27^.

The target hazard quotients (THQ) and the hazard index (HI) values are shown in Table 5.

**Table 5:** Target hazard quotients (THQ) and the hazard index (HI) values.

Species	THQ (Cd)	THQ (Hg)	THQ (Pb)	HI
European hake	0.00	0.38	0.00	0.38
Gilthead seabream	0.00	0.10	0.00	0.10
Patagonian squid	0.24	0.11	0.00	0.36
Sardine	0.00	0.12	0.00	0.13
Tuna fish	0.00	0.82	0.00	0.98
HI	0.25	1.53	0.00	

As it can be seen, the THQs were not exceeded for the tested heavy metals in the analysed species. However, there is a heightened risk for health effects due to the presence of mercury in seafood. Thus, we recommend that vulnerable segments of the population including young children, the elderly and pregnant women should limit the consumption of certain type of seafood, especially tuna in order to reduce possible adverse effects due to the presence of elevated levels of Hg. The results from our study are in good accordance with other studies which have also found heightened risk of adverse health effects when consuming certain types of seafood^48,49^.

## Conclusions

Our study points out that the population of the north-western part of Croatia could be at a heightened risks of adverse health effects due to the consumption of seafood with relatively high concentrations of Hg in some tested seafood species, namely tuna fish and European hake. To minimise the potential adverse effects that these heavy metals can have on human health we advise limiting the consumption of these fish species, especially when sensitive segments of the population such as children, the elderly and pregnant women are concerned. The results of our study strongly advocate for more rigours food quality control protocols to be implemented in the region.

## Data Availability

Data are available upon request and approval by the authors. The data can be requested from prof.dr.sc. Luka Traven, Department of Environmental Medicine, Medical faculty, University of Rijeka, Braće Branchetta 20/1, 51000 Rijeka, Croatia.
